# A Three-Phase Current Tacholess Envelope Order Analysis Method for Feature Extraction of Planetary Gearbox under Variable Speed Conditions

**DOI:** 10.3390/s21175714

**Published:** 2021-08-25

**Authors:** Xun Zhang, Guanghua Xu, Jiachen Kuang, Lin Suo, Sicong Zhang, Umair Khalique

**Affiliations:** 1 School of Mechanical Engineering, Xi’an Jiaotong University, Xi’an 710049, China; windofson@stu.xjtu.edu.cn (X.Z.); kjc1331@stu.xjtu.edu.cn (J.K.); suolin@stu.xjtu.edu.cn (L.S.); zhsicong@mail.xjtu.edu.cn (S.Z.); khalique_umair@stu.xjtu.edu.cn (U.K.); 2 State Key Laboratory for Manufacturing Systems Engineering, Xi’an Jiaotong University, Xi’an 710049, China

**Keywords:** planetary gearbox, tacholess, feature extraction, envelope order analysis, variable speed

## Abstract

Planetary gearboxes are the key components of large equipment, such as wind turbines, shield machines, etc. The operating state of the planetary gearbox is related to the safety of the equipment as a whole, and its feature extraction technology is essential. In assessing the problem of the non-stationarity of the current signal under variable speed conditions and the difficulty of evaluating the operating state of the planetary gearbox under a tacholess condition, a three-phase current, variable-speed tacholess envelope order analysis method is proposed. Firstly, a tacholess rotation speed estimation is completed by extracting the trend term of the instantaneous frequency of the asynchronous motor’s three-phase currents. The motor slip rate is assumed to be constant. Then, the envelope order analysis signal is obtained by re-sampling in the angular domain. Finally, the features of the envelope order signal are extracted, and a linear discriminant analysis (LDA) algorithm is used to fuse multiple indexes to generate a comprehensive feature reflecting the operating status of the planetary gearbox. The results of the simulation analysis and experimental verification show that the proposed method is effective in evaluating the operating state of the planetary gearbox under variable speed conditions. Compared with the traditional time–frequency ridge extraction method, the tacholess speed estimation method can improve the instantaneous speed estimation accuracy. The comprehensive index of envelope order completes the planetary gearbox state identification process, and a 95% classification accuracy rate is achieved.

## 1. Introduction

Gearboxes are used to transmit torque and power, and are widely used in industrial machinery. Among the various types of gearboxes, planetary gearboxes have the advantages of a large transmission ratio, strong bearing capacity, stable operation, and compact structure. They are widely used in industries or fields of operations such as wind turbines, aviation, ships, and mining. However, due to their frequent operation under the severe conditions of low speed and heavy load, planetary gearbox components wear and break from time to time. In addition, due to the speed fluctuations of the operating conditions, the dynamic signal of the planetary gearbox presents clear non-stationarity. Therefore, monitoring and diagnosing the planetary gearbox are extremely difficult. For example, one wind turbine gearbox in Denmark suffered a serious crack failure during operation, but its monitoring and diagnosis system failed to detect the failure in time, causing serious economic loss [1]. Studies on the state feature extraction technology of planetary gearboxes are of great significance to avoiding economic loss and improving safe production.

At present, vibration analysis is the main method for extracting the state features of a planetary gearbox. To achieve feature extraction of the vibration signal of a planetary gearbox, various signal processing methods have been used, such as envelope analysis [2], stochastic resonance [3], Hilbert Transform [4], time domain analysis [5], wavelet transform [6,7], empirical mode decomposition [8,9,10], etc. Lei Yaguo et al. [11] summarized and evaluated the planetary gear fault diagnosis method based on vibration analysis, showing that the vibration signal of the planetary gearbox has many congenital defects, making signal analysis difficult. For example, due to the complex vibration transmission path, the quality of the vibration signal is greatly affected by the measuring location of the sensor. On account of the multi-mode confusion of lateral vibration and the serious coupling of meshing vibration, the vibration signal has nonlinear characteristics. In addition, many experimental studies have shown that the frequency spectrum of vibration signals is asymmetrical, and noise pollution in the low-frequency region is serious, which also introduces great difficulties to signal feature extraction.

The vibration signal method suffers from sensitivity to measurement point location selection and complex spectrum law, and so scholars have used other types of signals to describe the state characteristics of the planetary gearbox, such as acoustic emission [12]. Because acoustic emission detection is a contact measurement method, the acoustic emission sensor needs to be close to the ring gear when installed, and so it is difficult to use.

Motor current signature analysis (MCSA) was proposed by Cardoso and Saraiva [13] in 1991 and used for motor fault diagnosis. It is a drive system fault diagnosis method oriented towards the stator current signal of the drive motor. This method focuses on the torsional vibration characteristics of the system, and can reflect the torsional vibration component in the power transmission path [14]. Compared with the vibration signal, the torsional vibration transmission path of the current signal is short, the sensor is easy to install, the low-frequency response is good, and the information collection approach is safe, making it suitable for industrial field machinery. Henao et al. [15] discussed the rationality of using the motor current method for torsional vibration monitoring, highlighting that the motor current sensor is easy to install and suitable for industrial field use. Zhang et al. [16] established an electric mechanics model of the motor–drag system, and verified the effectiveness of the motor current method in the diagnosis of planetary gearbox faults through simulation, finding that the current signal undergoes a more complex modulation phenomenon. Zhang et al. [17] completed fault diagnosis by extracting the time domain index of the current signal envelope for a planetary gearbox driven by a three-phase synchronous motor, but this could not be used to complete an automatic evaluation of the gearbox operating state. Shi Xianjiang et al. [18] used MCSA to reflect fault characteristics and used the empirical mode decomposition (EMD) method to identify characteristic frequency.

Traditional current analysis methods are usually used for single-channel current analysis. Three-phase motors are widely used in industrial equipment, and three-phase current signals can provide more information than single-phase signals. The most common method of fusion analysis of the three-phase current–phase relationship is the Park vector method [19]. The basic idea is to transform the three-phase current into a two-phase current through coordinate transformation, and complete fault diagnosis via a phase diagram of the two-phase current [13]. Cruz et al. [19] devised a comprehensive index for assessing the degree of motor failure through the phase diagram, and used it to evaluate the motor operating state. Pires et al. [20] used principal component analysis (PCA) to fuse three-phase current signals, and used the characteristic values of the principal components of the current as indicators to evaluate the operating status of motors with broken rotor bars, obtaining better results than with traditional single-phase current spectrum analysis.

The traditional monitoring and diagnosis methods are mostly based on signal feature extraction performed under uniform speed conditions. However, planetary gearboxes in industrial equipment often work under variable speed conditions [21]. Order analysis is the most commonly used signal analysis method under variable speed conditions [22]. The key approach of order analysis is to obtain the key-phasor signal reflecting the shaft’s instantaneous speed, and use it to complete angular domain resampling. The key-phasor signal can be directly collected by sensors, such as those monitoring speed and rotation angle. However, for equipment without pre-installed angle encoders, installing a speed sensor will be inconvenient [23].

The basic idea of tacholess order analysis technology is to extract the characteristics of the analysis signal and calculate the corresponding key-phasor signal. At present, mainstream tacholess order analysis methods mostly take the vibration signal as the object and extract the speed ridge as the instantaneous rotation speed signal through a specific image recognition algorithm to complete the order analysis. Wang Yi et al. [24] estimated the instantaneous frequency based on the cost function and used generalized Fourier transform to correct the time–frequency ridge. Experiments show that this method can extract more accurate time–frequency ridges than traditional methods under conditions of large rotational speed fluctuations. Etien et al. [25] proposed a method that estimates tacholess order tracking on a permanent magnet synchronous generator from a single current signal. In addition to vibration signals, sound signals, currents and video streams are also used for tacholess speed estimation [26].

For state feature extraction under variable speed conditions, traditional time domain and frequency domain analysis methods are no longer applicable. In recent years, with the rise of artificial intelligence and machine learning, research related to these issues has gradually increased, such as that focusing on neural networks and support vector machines [27,28,29]. The basic idea is to extract appropriate indicators based on the empirical characteristics of the variable speed signal, and classify the indicators to achieve fault diagnosis. Compared with traditional methods, the above methods are more intelligent and less dependent on experience.

Aiming at the problem of the non-stationarity of the current signal under variable speed conditions and the difficulty of evaluating the operating state of the planetary gearbox under tacholess conditions, this paper proposes a three-phase current variable-speed tacholess envelope order analysis method. This method completes the instantaneous rotation speed estimation via the three-phase current signals. The order analysis of the envelope signal is completed through angular domain resampling. By extracting the features of the envelope order signal, the linear discriminant analysis technique is used to fuse multiple indicators to form a comprehensive reflection of the characteristics of planetary gearbox operating conditions. Through simulation analysis and experimental verification, the proposed method for evaluating the operating state of a planetary gearbox under variable speed conditions is proven to be effective.

The rest of this article is structured as follows. In Section 2, the proposed method is introduced. Its effectiveness is verified by simulation analysis and experiments in Section 3. The conclusions are drawn in Section 4.

## 2. Proposed Method

### 2.1. Framework

The instantaneous phase of the current signal under variable speed conditions is no longer linear, meaning the current signal will display clear non-stationarity. At this time, traditional stationary signal analysis methods are no longer applicable, and order analysis is the best method for variable speed signal analysis. This method can overcome frequency leakage, ensure the validity of historical data, and extract power frequency components [30].

Tacholess signal analysis methods usually take vibration signals as their object, extract and estimate instantaneous rotation speed through the time–frequency ridgelines, and complete order analysis [31]. The signal processing flow [31] is as follows. Firstly, a short-time Fourier transform (STFT) is performed on the vibration signal to obtain the time–frequency diagram. Secondly, a speed ridge reflecting the instantaneous speed information of the system is derived from the time–frequency diagram, and then the appropriate image processing algorithm is used to extract the speed ridge and obtain the instantaneous speed. Finally, tachometer information is obtained through the instantaneous speed, and order analysis is completed by re-sampling in the angular domain.

The instantaneous speed estimation is the most critical part. A significant problem of existing tacholess signal analysis methods is that they struggle to accurately estimate the instantaneous speed. First of all, the main energy of the vibration signals of gearboxes, bearings, and other mechanical parts is not concentrated in the instantaneous frequency. The time–frequency ridges are usually weak and difficult to identify. Secondly, the speed signal usually contains other types of ridges and noise, which greatly affects speed ridge identification.

It can be seen from the above analysis that the effectiveness of tacholess order analysis depends on the signal quality and the accuracy of the instantaneous speed estimation. Since the energy of the motor current signal is concentrated on the fundamental frequency, it is less affected by noise interference. There is a phase relationship between the three-phase current signals, which provides very rich frequency conversion information. The above characteristics of the current signal accommodate the requirements of tacholess signal analysis.

**Figure 1:**
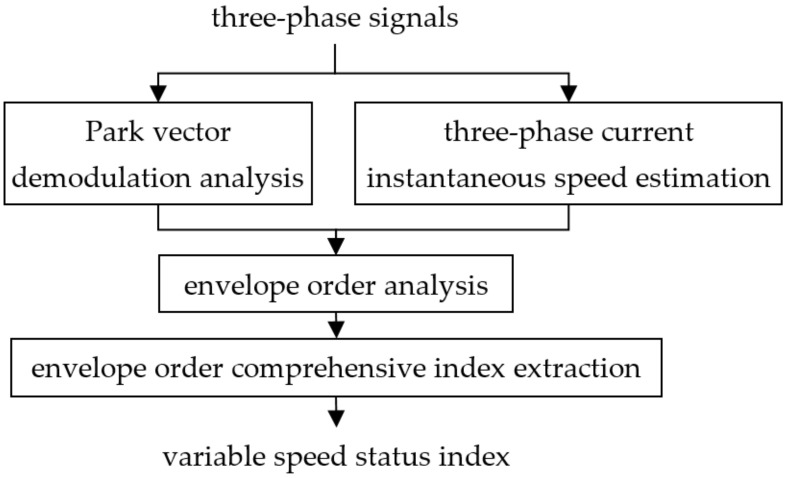
The procedure of tacholess current signal analysis of variable-speed planetary gearbox.

Based on the above-described basic ideas, this paper proposes a three-phase current tacholess order index extraction method to extract the features from the operating state of the planetary gearbox under variable speed conditions. The basic procedure is shown in Figure 1.

The main process of this method is as follows. Since all the torsional vibration information of the current signal is modulated around the fundamental frequency, the Park vector demodulation analysis method is used to obtain the instantaneous amplitude and instantaneous frequency of the three-phase current signals. For speed estimation under tacholess conditions, the proposed three-phase current instantaneous speed estimation algorithm calculates the instantaneous speed corresponding to the current acquisition time. Secondly, the instantaneous speed signal is used for angular domain re-sampling to obtain the instantaneous demodulated amplitude and instantaneous frequency signals. Then, the extraction of order signals can be completed. Finally, following the feature extraction of order signals and the envelope order comprehensive index extraction method, the characteristic information of the planetary gearbox operating state under variable speed conditions can be obtained.

The following sections will introduce the principles and algorithms used in each step of the above-described method.

### 2.2. Three-Phase Current Instantaneous Speed Estimation

In tacholess signal analysis, instantaneous speed estimation is important. For a transmission system, the instantaneous speed of the planetary gearbox can be calculated based on the motor speed and the transmission ratio. Therefore, only the instantaneous speed of the motor needs to be calculated. Under variable speed conditions, the fundamental frequency of the current signal has time-varying characteristics. For an asynchronous motor, assuming that $n\left(t\right)$ is the actual motor speed in RPM, p is the number of pole pairs of the motor, s is the motor slip rate, and $f_{e}\left(t\right)$ is the fundamental frequency of the current signal in Hz, then:

(1) $$n\left(t\right)=60\left(1-s\right)/p\cdot f_{e}\left(t\right)$$

The motor slip rate s is assumed to be constant in this manuscript if the motor is operating under relatively stable conditions.

It can be seen from Equation (1) that the key to instantaneous speed estimation is to calculate the instantaneous fundamental frequency $f_{e}\left(t\right)$ of the three-phase current signals. Since the main energy of the current signal is concentrated on the fundamental frequency, there is a relationship between the instantaneous fundamental frequency and the instantaneous frequency. According to the vibration signal model for a local fault in the planetary gearbox [32], the three-phase current signals of the variable speed condition and instantaneous shaft phase function can be expressed as

(2) $$\left\{\begin{array}{c} i_{U}\left(t\right)=\left(1+A_{m}\left(1+A_{f}\cos\left(f_{c0}\varphi \left(t\right)+\theta \right)\right)cosf_{m0}\varphi \left(t\right)\right)\cos\left(f_{e0}\varphi \left(t\right)\right)\\ i_{V}\left(t\right)=\left(1+A_{m}\left(1+A_{f}\cos\left(f_{c0}\varphi \left(t\right)+\theta \right)\right)cosf_{m0}\varphi \left(t\right)\right)\cos\left(f_{e0}\varphi \left(t\right)-2\pi /3\right)\\ i_{W}\left(t\right)=\left(1+A_{m}\left(1+A_{f}\cos\left(f_{c0}\varphi \left(t\right)+\theta \right)\right)cosf_{m0}\varphi \left(t\right)\right)\cos\left(f_{e0}\varphi \left(t\right)-4\pi /3\right) \end{array}\right.$$

(3) $$\varphi \left(t\right)=2\pi \int_{-\infty }^{t}f\left(\tau \right)d\tau$$

where $i_{U}$, $i_{V}$ and $i_{W}$ are the *U* phase, *V* phase and *W* phase current, respectively; $f_{e0}$, $f_{m0}$ and $f_{c0}$ are the current fundamental, meshing and characteristic frequency under unit rotation frequency, respectively, reflecting the order frequency of each; $A_{m}$ and $A_{f}$ are the modulated amplitude of the meshing frequency and the fault characteristic frequency, respectively; θ is the initial phase; $\varphi \left(t\right)$ is the instantaneous shaft phase function.

Using the Park vector demodulation analysis method [33], the current signal of the stationary coordinate system can be obtained:

(4) $$\left\{\begin{array}{c} i_{\alpha }\left(t\right)=\left(1+A_{m}\left(1+A_{f}\cos\left(f_{c0}\varphi \left(t\right)+\theta \right)\right)cosf_{m0}\varphi \left(t\right)\right)\cos\left(f_{e0}\varphi \left(t\right)\right)\\ i_{\beta }\left(t\right)=\left(1+A_{m}\left(1+A_{f}\cos\left(f_{c0}\varphi \left(t\right)+\theta \right)\right)cosf_{m0}\varphi \left(t\right)\right)\sin\left(f_{e0}\varphi \left(t\right)\right) \end{array}\right.$$

where $i_{\alpha }$ and $i_{\beta }$ are derived from $i_{U}$, $i_{V}$ and $i_{W}$ using Park transform at a 90° angle.

The instantaneous amplitude and instantaneous phase are:

(5) $$\left\{\begin{array}{c} A\left(t\right)=1+A_{m}\left(1+A_{f}\cos\left(f_{c0}\varphi \left(t\right)+\theta \right)\right)cosf_{m0}\varphi \left(t\right)\\ \theta \left(t\right)=f_{e0}\varphi \left(t\right) \end{array}\right.$$

The instantaneous frequency (the derivative of the instantaneous phase) is:

(6) $$f\left(t\right)=\theta^{\prime }\left(t\right)/2\pi =f_{e}\left(t\right)$$

Ideally, the instantaneous fundamental frequency of the current signal would be the same as the instantaneous frequency, as shown in Equation (6). Due to the presence of electromechanical coupling sites, instantaneous frequency signals will also have frequency components that reflect the characteristics of torsional vibration in actual electromechanical systems. Therefore, the instantaneous frequency of the current signal can be regarded as the superposition of the instantaneous fundamental frequency and torsional frequency components.

**Figure 2:**
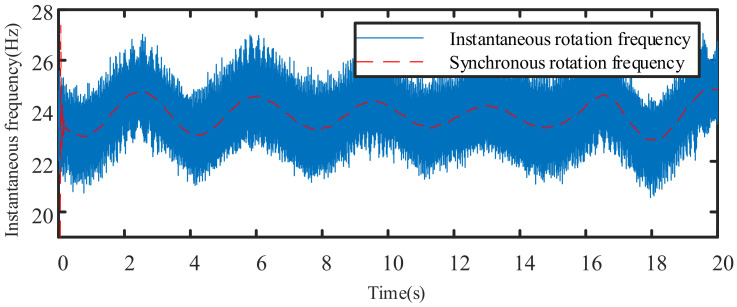
The relationship between instantaneous rotation frequency and synchronous rotation frequency.

Generally, the frequency of the torsional vibration signal in a variable-speed mechanical system is much greater than the change frequency of the instantaneous speed, so the instantaneous fundamental frequency appears as a low-frequency trend item in the instantaneous frequency. Figure 2 shows the relationship between the instantaneous rotation frequency and the synchronous rotation frequency of the motor during the operation of a specific planetary gearbox.

The problem of instantaneous speed estimation reduces to the extraction of the trend term of the current instantaneous frequency signal. Under the signal processing method, there are many ways to extract trend items. Obvious trends can be identified by function fitting, periodic trends can be obtained through spectral analysis, and implicit trends must be processed by empirical mode decomposition, wavelet packet decomposition, or time series analysis. According to the characteristic frequency of the current signal and the frequency of the rotational speed fluctuation, low-pass filtering can be used to extract the low-frequency signal as the instantaneous synchronous rotation frequency.

**Figure 3:**
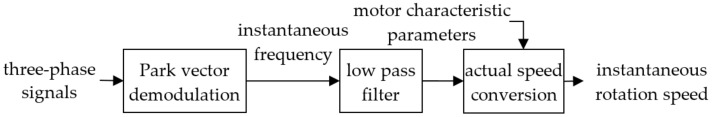
Three-phase current instantaneous rotation speed estimation algorithm.

In summary, the three-phase current instantaneous rotation speed estimation algorithm proposed in this section is shown in Figure 3.

Since the instantaneous phase and the instantaneous frequency display a differential relationship, estimation of the instantaneous rotation angle of the shaft requires the instantaneous phase, which is derived from the Park vector demodulation method in the algorithm process shown in Equations (4)–(6). After subsequent operations, the instantaneous angle of rotation can be estimated.

### 2.3. Envelope Order Analysis

After the instantaneous speed is estimated, the analysis of non-stationary signals can be completed by order analysis. In this section, according to the characteristics of the current signal, the traditional order analysis method will be improved, and the envelope order analysis method of the three-phase current signals will be obtained.

**Figure 4:**
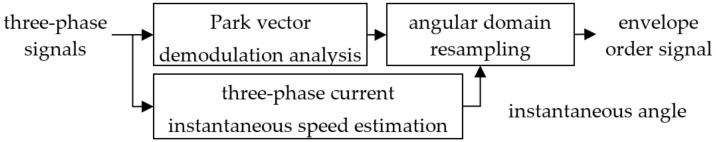
The flow of envelope order algorithm.

Order analysis is a method of transforming the time domain signal into the angular domain. Because the fault characteristic frequency of the current signal is modulated around the signal, it is necessary to demodulate the current signal before performing order analysis. The Park vector demodulation analysis method [33] is used to extract the instantaneous amplitude and instantaneous frequency of the three-phase current signal for analysis. At the same time, according to the instantaneous speed estimation algorithm proposed in the previous section, the instantaneous rotation angle signal can be obtained from the three-phase current signal so as to complete angular domain resampling. Therefore, the flow of the envelope order analysis method in this section is shown in Figure 4.

There are two significant differences between the envelope order analysis method employed in this paper and the traditional order analysis method. Firstly, the order analysis in this paper is based on the current envelope signal, and the acquisition of the envelope signal depends on the three-phase current analysis method. Secondly, the traditional instantaneous rotation speed estimation method must calculate the instantaneous phase of the shaft through instantaneous frequency integration, but the instantaneous rotation speed extraction algorithm proposed in this paper can calculate the instantaneous phase while demodulating the three-phase current, avoiding repeated integral calculation.

### 2.4. Envelope Order Comprehensive Index Extraction Algorithm

According to the basic theoretical characteristics of order analysis, the envelope order signal calculated by the above process is equivalent to the variable speed as calculated by the steady process. For the analysis of envelope order signals, stationary signal analysis methods, such as spectrum analysis, can be used. Regarding the extraction of the state features of the planetary gearbox under uniform speed conditions, theoretical analysis and experiments show that the entropy and kurtosis coefficient of the demodulated signal can be used as indicators to reflect the characteristics of the planetary gearbox’s operating state [33]. Therefore, in this section, the entropy, spectral entropy, kurtosis coefficient, and spectral kurtosis coefficient of the envelope order signal are extracted as features.

Under constant speed conditions, each feature extracted can be used to reflect the operating state of the planetary gearbox. However, under variable speed conditions, due to the influence of speed fluctuations, a single index is not sufficient to summarize all the motion characteristics of the planetary gearbox. Therefore, it is necessary to use certain feature dimensionality reduction and fusion methods to fuse multiple indicator features and thus obtain representative comprehensive indicators. In the field of machine learning, commonly used feature dimensionality reduction methods include principal component analysis (PCA) and linear discriminant analysis (LDA). In contrast, PCA does not require prior label information, and dimensionality reduction and classification are based on the variance of the feature data set, while LDA can use prior label information to focus on the mean difference between features in different classes. In contrast, LDA can obtain more accurate results because of its prior label information. Therefore, this paper chooses LDA as the feature dimensionality reduction method, as it can provide a linear combination of the extracted feature indicators, namely:

(7) $$y=\overset{n}{\underset{i=1}{\sum }}\omega_{i}x_{i}$$

where $x_{i}$ is the *i*-th feature; n is the number of features; y is the comprehensive index; $\omega_{i}$ is the weighting coefficient of the *i*-th index to the comprehensive index.

The principle of the LDA algorithm is to find the best coefficient combination $\omega_{i}$ through training samples. The LDA algorithm uses samples of the same class compactly after classification, and samples from different classes discretely. The criterion function used is:

(8) $$J_{F}\left(\omega \right)=\frac{{\left({\tilde{m}}_{1}-{\tilde{m}}_{2}\right)}^{2}}{{\tilde{S}}_{1}^{2}+{\tilde{S}}_{2}^{2}}$$

where ${\tilde{m}}_{i}$ is the mean value of the *i*-th class after dimensionality reduction; ${\tilde{S}}_{i}$ is the intra-class divergence after dimensionality reduction.

The right side of Equation (8) is not an explicit function of the normal vector *ω*. The above formula needs transformation to solve the problem. The mean vector of the two types of samples is

(9) $$m_{i}=\frac{1}{n}\cdot \underset{x_{j}\in \Omega_{i}}{\sum }x_{j}\;\;\;i=1,2$$

where $\Omega_{i}$ is the *i*-th class.

For the description of the closeness between samples of the same class, the intra-sample divergence is introduced:

(10) $$S_{w}=\underset{i=1,2}{\sum }\underset{x_{j}\in \Omega_{i}}{\sum }\left(x_{j}-m_{i}\right){\left(x_{j}-m_{i}\right)}^{T}$$

For the description of the dispersion of samples between Class 1 and Class 2, the inter-sample divergence is introduced:

(11) $$S_{b}=\left(m_{1}-m_{2}\right){\left(m_{1}-m_{2}\right)}^{T}$$

Equations (9)–(11) are incorporated into Equation (8), which gives:

(12) $$J_{F}\left(\omega \right)=\frac{\omega^{T}S_{w}\omega }{\omega^{T}S_{b}\omega }$$

In order to find the maximum value of *J_F_*(*ω*), the partial derivative of *ω* should be obtained:

(13) $$\frac{\partial J_{F}\left(\omega \right)}{\partial \omega }=\frac{\omega^{T}S_{w}\omega }{{\left(\omega^{T}S_{b}\omega \right)}^{2}}\left(S_{b}\omega -J_{F}\left(\omega \right)S_{w}\omega \right)$$

The partial derivative is set to 0 and the stationary point is found, that is:

(14) $$S_{b}\omega =J_{F}\left(\omega \right)S_{w}\omega$$

If this gives *J_F_*(*ω*) = *λ*, then the above problem is transformed into the following generalized eigenvalue problem:

(15) $$S_{w}^{-1}S_{b}\omega =\lambda \omega$$

Therefore, the above problem can be transformed into an eigenvalue decomposition problem. By solving the eigenvector, the *ω* can be obtained.

**Figure 5:**
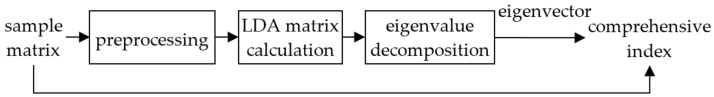
LDA algorithm flow.

The basic flow of the LDA algorithm is shown in Figure 5.

**Figure 6:**
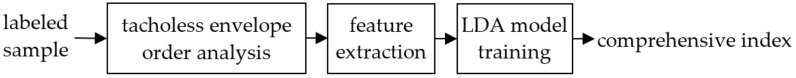
Model training for comprehensive index extraction.

To extract the comprehensive indicators, it is first necessary to train the sample model with prior information to obtain the formula for calculating the comprehensive indicators. The labeled sample signals are collected under two typical conditions: normal and abnormal. The sampling conditions of the sample signals can be changed in any order within the predicted speed fluctuation range of the planetary gearbox. The instantaneous frequency and instantaneous amplitude and feature extraction are calculated by the tacholess envelope order analysis method. The extracted features and label information are put into the LDA model for training, and finally, a comprehensive index calculation formula can be obtained. The process of model training is shown in Figure 6.

Through model training, the weighting coefficient $\omega_{i}$ of the index can be obtained, and the comprehensive index expression shown in Equation (7) can accordingly be obtained. Since the model is trained on variable speed signals, the obtained comprehensive index can also be applied in the state feature extraction for the variable speed planetary gearbox. At this time, for a group of variable-speed three-phase current signals, the extracted envelope signal characteristics and the characteristics of the planetary gearbox operating state can be obtained through tacholess envelope order analysis and from Equation (7), respectively, under variable-speed conditions.

## 3. Simulation Signal Analysis and Experiment Verification

### 3.1. Simulation Signal Analysis

**Figure 7:**
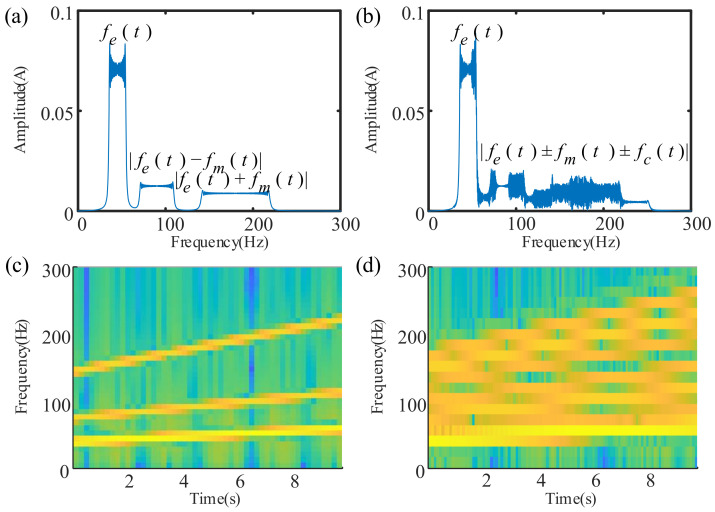
Spectrum and time–frequency diagram of the simulated signal under variable speed conditions: (**a**) spectrum of normal gearbox; (**b**) spectrum of abnormal gearbox; (**c**) time–frequency spectrum of normal gearbox; (**d**) time–frequency spectrum of abnormal gearbox.

This section will verify the effectiveness of the proposed algorithm through data simulation analysis. In the variable speed current signal model of Equation (2), we take a meshing frequency order $f_{m0}=3.0$, an abnormal characteristic frequency order $f_{c0}=0.6$; the modulation amplitude of the meshing frequency $A_{m}=0.5$, the modulation amplitude of the abnormal characteristic frequency $A_{f}=1.0$. We suppose that the instantaneous rotation frequency of the drive motor in the variable speed process is a rotation process that uniformly accelerates from 35 Hz to 55 Hz. The sampling frequency is 1024 Hz. The frequency spectrum and time–frequency characteristics of the simulated current signal of the normal gearbox are shown in Figure 7a,c, and those of the abnormal gearbox are shown in Figure 7b,d. The STFT parameters used for the normal gearbox shown in Figure 7c are as follows. The window size is 256, the number of overlapped samples is 64, the number of DFT points is 128, and the acquisition frequency is 1024 Hz. The STFT parameters used for the abnormal gearbox in Figure 7d are as follows. The window size is 128, the number of overlapped samples is 32, the number of DFT points is 64, and the acquisition frequency is 1024 Hz.

It can be seen from Figure 7a that the normal gearbox’s current signal contains a series of components that characterize the fundamental frequency of the current signal in the 35–55 Hz frequency band, and it contains meshing frequency components modulated by the fundamental frequency at around 100 Hz. However, because spectrum analysis does not address time resolution, it is difficult to reflect the relationship between frequency and time. In addition, modulation characteristics cannot be reflected. It can be seen from Figure 7b that there are multiple aliasing frequency components at 100–300 Hz. If one only consults the frequency spectrum, its source cannot be clearly identified, let alone its fault.

In the time–frequency figures, the characteristics of the instantaneous speed changing with time can be observed. In Figure 7c,d, straight lines that reflect the instantaneous fundamental frequency of the current (i.e., the speed ridge) are shown. However, it is difficult to obtain time–frequency ridges from this image. Because the spectral resolution is very low, only the approximate range of the instantaneous frequency can be obtained, and its accuracy is not high. This introduces great difficulty into the order analysis.

In addition, from the comparison of Figure 7c,d, it can be seen that the normal gearbox’s current signal contains three time–frequency ridges: the current fundamental frequency, the meshing frequency modulated by the fundamental frequency, and the time–frequency ridge of the abnormal characteristic frequency. However, due to the low-frequency resolution, the fault characteristic frequency ridgeline and the surrounding meshing frequency ridge line are seriously aliased, and fault identification cannot be completed.

**Figure 8:**
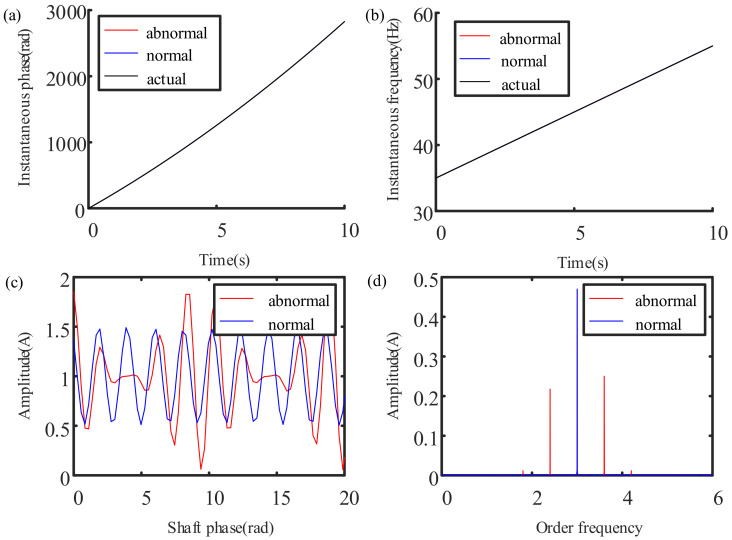
Results of order analysis via Park vector demodulation method for the simulated signal: (**a**) instantaneous phase; (**b**) instantaneous frequency; (**c**) angle waveform; (**d**) order spectrum.

The results of the proposed method in this manuscript are shown in Figure 8. Figure 8a,b, respectively, show the instantaneous phase and instantaneous frequency of the signal as derived by this method. It can be seen that the calculated value is very close to the actual value. Figure 8c shows the waveform of the current instantaneous amplitude of the normal and abnormal gearboxes through angular domain re-sampling. It can be seen that the normal gearbox exhibits a steady-state sine law, and the abnormal gearbox exhibits a modulation law. The frequency spectrum analysis is performed on the angular domain signal to obtain the order spectrum, and this is shown in Figure 8d. This shows one order (3.0) reflecting the meshing frequency on the analog normal signal order spectrum, and we can also see a modulation of the meshing frequency order to the abnormal order (0.6) around the analog abnormal signal. After order analysis, the original variable speed process is equivalent to a uniform speed process, and the previous steady signal analysis method can be used for processing to complete the state feature extraction.

From the above simulation results, it can be seen that the three-phase current signal under variable speed tacholess working conditions is non-stationary, and the frequency spectrum appears as a “frequency blur”, meaning effective analysis cannot be completed. Although time–frequency analysis can identify the non-stationarity of the signal and qualitatively determine the changing trend of the speed, it is restricted by time–frequency resolution, and cannot complete an effective instantaneous speed calculation. Using the three-phase current envelope order analysis method proposed in this paper, the instantaneous rotation speed can be estimated by the current signal, and the operating status of the planetary gearbox can be identified through order analysis. In Figure 7c,d, it is difficult to identify the time–frequency ridge, or the abnormality. In Figure 8b, the instantaneous frequency is extracted effectively and clearly. The method proposed in this paper performs better than the traditional time–frequency ridge extraction method in estimating instantaneous speed.

### 3.2. Experimental Verification of Three-Phase Current Instantaneous Speed Estimation Slgorithm

This section will verify the effectiveness of the instantaneous speed estimation algorithm applied to the three-phase current signal. An experiment on the speed change in the planetary gear platform was performed. The experimental process was as follows.

#### 3.2.1. Experimental Setup

**Figure 9:**
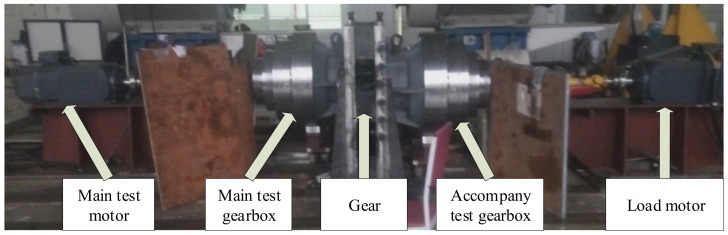
The experimental setup.

The experimental setup is shown in Figure 9. The gearbox model is QHPD110T, which employs four-stage planetary gear transmission. The main test motor is a three-phase asynchronous motor with a pole pair number of 2 and a power of 79 kW. There are two identical planetary gearboxes: the main tester, and the accompanying tester. They are connected back to back by a pair of large gears. The test gearbox is connected to the generator to recycle and utilize the electric energy. The current sensors used are Fluke i200s clamp-type. The sampling frequency is 2048 Hz, and the sampling time length is 100 s.

#### 3.2.2. Experimental Results and Analysis

**Figure 10:**
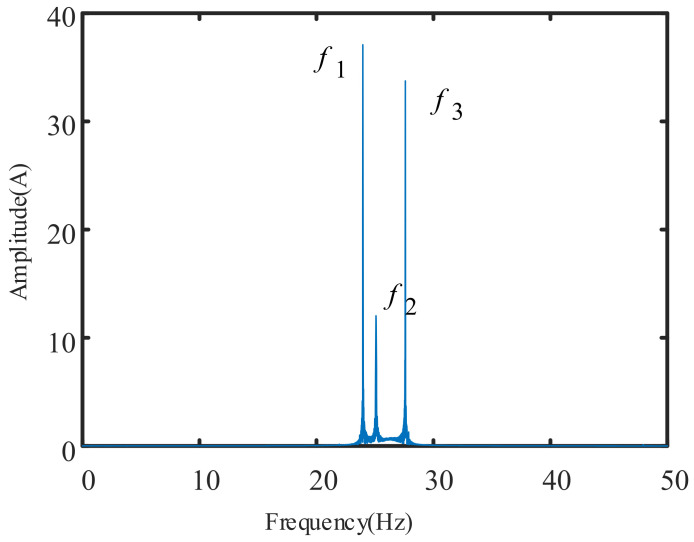
The frequency spectrum of the single-phase current signal of the variable-speed planetary gearbox.

First, the planetary gearbox speed is set and held at $n_{1}$, which is around 1440 rpm (24 Hz). Then, the speed is increased to $n_{2}=1.05n_{1}$ until stable. Finally, the speed is adjusted to $n_{3}=1.15n_{1}$ until stable. The experimental process can simulate the variable speed operation of a planetary gearbox under certain field conditions. The three-phase motor stator current signals are collected during the experiment. The spectrum analysis of the single-phase current is as shown in Figure 10.

The frequency spectrum shown in Figure 10 contains three frequency components, $f_{1}=23.97$ Hz, $f_{2}=25.09$ Hz, and $f_{3}=27.60$ Hz, which correspond to the three uniform speed operation stages of the planetary gearbox during variable-speed operation. However, if the non-stationary characteristics of the signal are ignored, it may not be understood that $f_{1}$ is the fundamental frequency of the current signal and $f_{2}$ and $f_{3}$ represent the characteristic frequency of a certain fault. Therefore, the operating state of the planetary gearbox may be misjudged.

**Figure 11:**
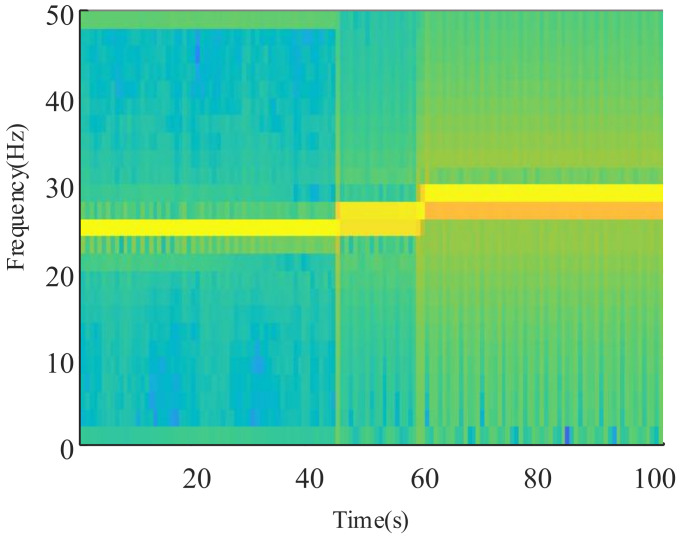
Time–frequency diagram of the current signal of a variable-speed planetary gearbox.

Since the current signal appears asymmetrically in the frequency spectrum of Figure 10, it is reasonable to assume that it reflects a variable speed condition. To verify this, the current signal is transformed by short-time Fourier transform, and a time–frequency diagram is obtained as shown in Figure 11. The parameters used for the STFT are as follows. The window size is 1024, the number of overlapped samples is 256, the number of DFT points is 512, and the acquisition frequency is 2048 Hz.

The current signal appears non-stationary in the time–frequency diagram, as shown in Figure 11. It can thus be shown through time–frequency analysis that the planetary gearbox is operating under variable speed conditions. The deep line near 25 Hz in Figure 11 represents the time–frequency ridgeline of this variable speed process. The change in the speed ridge shows that the entire speed change process generally includes three uniform speed stages and two acceleration stages. However, as this process is limited by the frequency resolution, it is impossible to accurately obtain the rotation frequency of each stable operating state. Furthermore, as it is also limited by the time domain resolution, it is also impossible to accurately obtain the starting time of each acceleration process. Due to the Heisenberg uncertainty principle, we cannot determine the exact motion law of each acceleration process. Therefore, although the time–frequency analysis method can roughly determine the speed variation law of the variable speed signal, it cannot quantitatively and accurately extract the instantaneous rotation speed.

**Figure 12:**
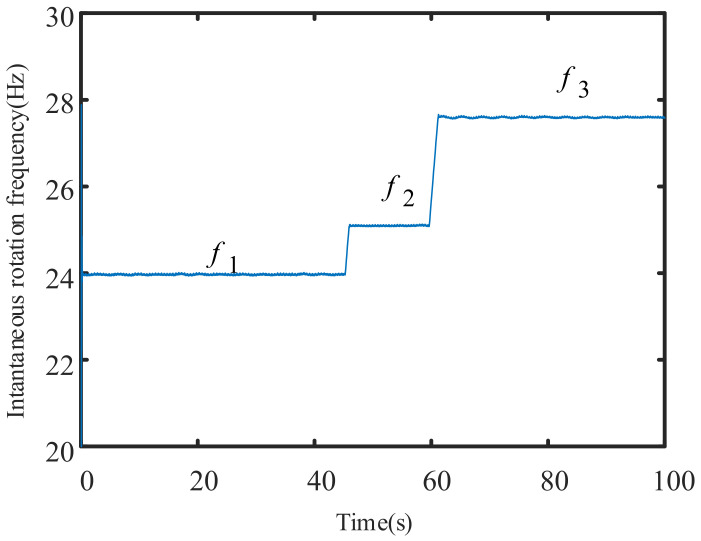
The instantaneous rotation frequency conversion is extracted by the method proposed in this manuscript.

As such, the three-phase current instantaneous rotation speed estimation algorithm proposed in this paper is applied to the above signals, and the instantaneous rotation frequency is obtained, as shown in Figure 12.

The instantaneous rotation frequency visualization in Figure 12 shows the entire variable speed process. The three horizontal lines represent the three steady-state operation stages, and the two oblique lines represent the operation law of the acceleration process. The ordinate values of the three horizontal = segments are $f_{1}=23.97$ Hz, $f_{2}=25.09$ Hz and $f_{3}=27.60$ Hz, which correspond to the three frequency peaks of the frequency spectrum in Figure 10, as well as the three experimental design speeds $n_{1}$, $n_{2}$, and $n_{3}$. This shows that the three-phase current instantaneous speed extraction method proposed in this paper is effective.

### 3.3. Experimental Verification of Tacholess Envelope Order Analysis

In this section, a single-stage planetary gear test bench will be used for a planetary gearbox variable speed fault diagnosis experiment, in order to verify the three-phase current signal tacholess envelope order analysis method proposed in this research.

#### 3.3.1. Experimental Setup

**Figure 13:**
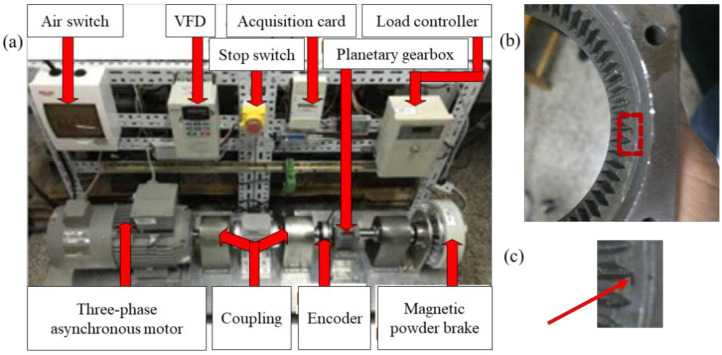
The experimental setup: (**a**) the planetary gear test bench; (**b**) gear ring with root crack fault; (**c**) enhanced image of (**b**).

The experimental setup is shown in Figure 13. The planetary gear test bench is driven by a three-phase asynchronous motor; the number of pole pairs is 2, and it has a power of 52 W. The model of the planetary gear is RUINO TRANSMISSION pxds90-3, and the numbers of the sun gear, planetary gear and ring gear are 36, 18, and 72, respectively. The number of planetary wheels is 3. The test bench contains planetary gearboxes with normal and cracked ring gears. The ring gear’s crack is 1 mm wide and penetrates into 1/3 of the depth of the gear; it is located on the root of the ring gear and is introduced by wire cutting. The acquisition frequency is 2560 Hz, and the sampling time is 100 s.

#### 3.3.2. Experimental Results and Analysis

In the experiment, normal and faulty planetary gearboxes are operated under variable speed conditions, and the current signals of the three-phase motor are collected. The tacholess envelope order analysis method is used to derive the envelope order spectrum, and fault judgment is determined via the difference of the envelope order spectrum.

In the experiment, to control the variable-speed operation of the planetary gearbox, the output frequency of the inverter is continuously adjusted at intervals of 0.1 Hz to change the output speed of the motor. Due to the randomness of inverter adjustment, the experiment reflects a general variable-speed process. Correspondingly, the three-phase current signals have the general characteristics of a non-stationary signal.

Through structural analysis of the planetary gearbox, the order characteristic frequency of the planetary gearbox is calculated, as shown in Table 1.

**Figure 14:**
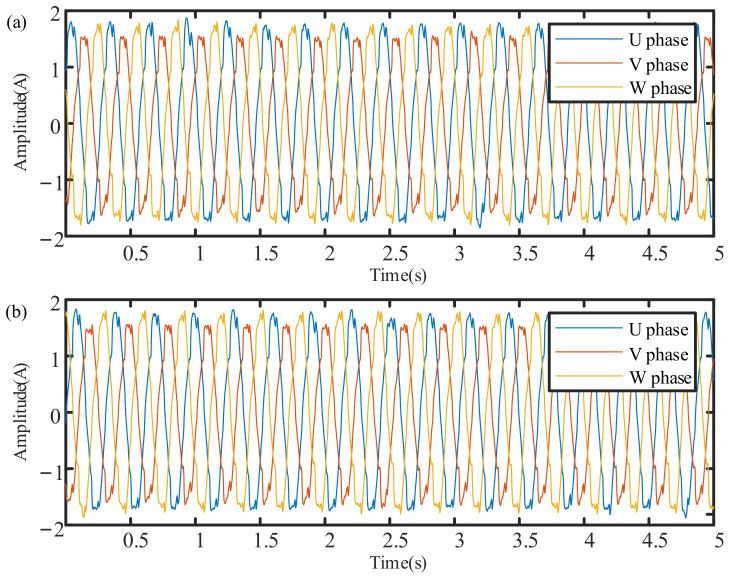
Original current signals collected from the planetary gearbox: (**a**) U phase, V phase, and W phase current of the normal gearbox; (**b**) U phase, V phase, and W phase current of the faulty gearbox.

The first 5 s of the three-phase current waveform collected from the normal planetary gearbox is shown in Figure 14a, and those for the faulty gearbox are shown in Figure 14b.

**Figure 15:**
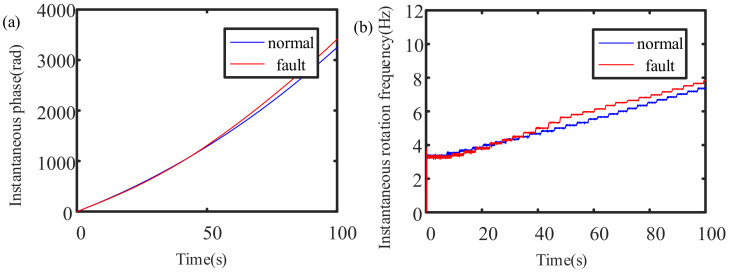
Instantaneous rotation frequency of the experimental gearbox: (**a**) instantaneous phase; (**b**) instantaneous rotation frequency.

The instantaneous rotation speeds of the three-phase current signals of the normal and faulty planetary gearboxes are estimated. The extracted instantaneous phase and instantaneous rotation speed signals are shown in Figure 15.

Figure 15b clearly shows that the instantaneous rotation frequency is subject to a stepwise increasing change law, and the increment is 0.1 Hz each time. Since the rotation frequency in the experiment is also increased stepwise at intervals of 0.1 Hz, the experimental results are consistent with the theoretical analysis, which also confirms that the instantaneous rotation frequency extraction algorithm proposed in this paper can be used for the instantaneous rotation frequency estimation of the variable-speed planetary gearbox.

**Figure 16:**
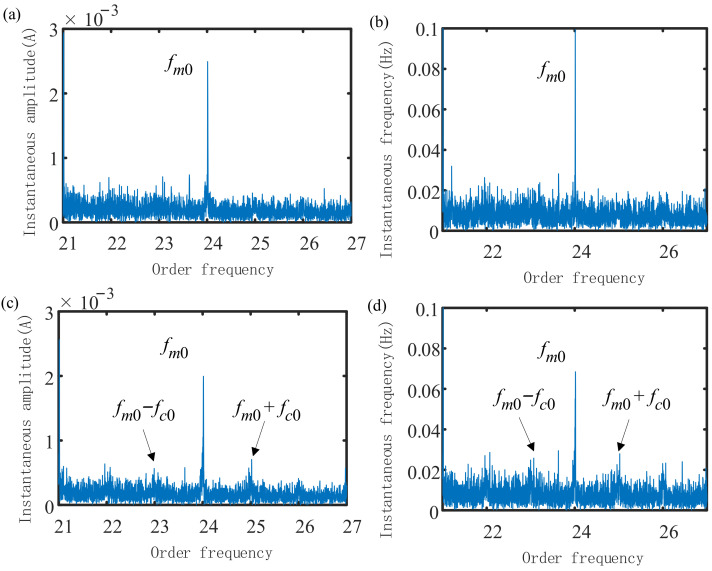
Envelope order spectrum of normal and faulty gearboxes: (**a**) envelope order amplitude of normal gearbox; (**b**) envelope order frequency of normal gearbox; (**c**) envelope order amplitude of faulty gearbox; (**d**) envelope order frequency of faulty gearbox.

The envelope order spectra of normal and faulty planetary gearboxes are shown in Figure 16.

The envelope order amplitude of the normal gearbox only contains the meshing frequency order ($f_{m0}$), while the faulty gearbox also contains a ring gear-concentrated fault order modulated by the meshing frequency ($f_{m0}\pm f_{c0}$) and its sidebands, as shown in Figure 16c,d. However, because the gearbox failure is relatively weak, the fault characteristic order is not obvious.

In summary, the tacholess order analysis method proposed in this paper can be applied in the state feature extraction of planetary gearboxes under variable speed conditions. Sidebands appear on the envelope order signal under faulty conditions, making the spectrum structure near the meshing order more complicated. To this end, we use index fusion technology to calculate the comprehensive index reflecting the operating state of the planetary gearbox.

### 3.4. Validation of the Extraction Algorithm for the Comprehensive Index of the Envelope Order

In this section, we will verify the effectiveness of the algorithm for extracting the comprehensive index of the envelope order via the operation state evaluation experiment of the variable-speed planetary gearbox. The experimental setup described in Section 3.3 is used. The normal and faulty planetary gearboxes are respectively subjected to 50 variable speed signal acquisition experiments. The signal acquisition parameters of each experiment are kept consistent to ensure that the indicators do not differ as a result of the length of the data. The speed range of the planetary gearbox is 200 rpm (3.33 Hz) to 400 rpm (6.67 Hz), and the motor output speed is randomly changed through the inverter, meaning the speed change rule of each experiment is different.

**Figure 17:**
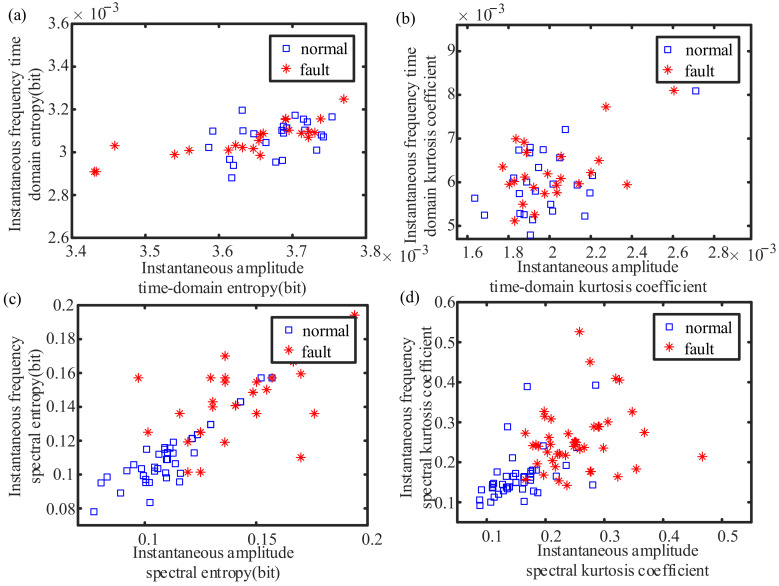
The envelope order index of the experimental gearbox: (**a**) time domain entropy; (**b**) time domain kurtosis coefficient; (**c**) spectral entropy; (**d**) spectral kurtosis coefficient.

Tacholess envelope order analysis is performed on the collected three-phase current signals. The time domain entropy of the angular domain’s instantaneous amplitude, the time domain entropy of the angular domain’s instantaneous frequency, the spectral entropy of the angular domain’s instantaneous amplitude, the spectral entropy of the angular domain’s instantaneous frequency, the time domain kurtosis coefficient of the angular domain’s instantaneous amplitude, the time domain kurtosis coefficient of the angular domain’s instantaneous frequency, the spectral kurtosis coefficient of the angular domain’s instantaneous amplitude, and the spectral kurtosis coefficient of the angular domain’s instantaneous frequency are taken as the characteristic values of each group of current signals. A feature vector composed of eight state features can be obtained. The sample characteristics of 100 experiments are shown in Figure 17.

There are certain differences in the envelope order characteristics of normal and faulty planetary gearboxes, as shown in Figure 17. However, due to the relatively weak failure of planetary gearboxes, a single index is not sufficient to distinguish between normal and faulty planetary gearboxes. Therefore, each feature is fused, and a comprehensive index is proposed through model training.

From the 50 sets of normal sample data, 40 are randomly selected, and the same is done for the faulty samples. Then, training samples composed of 80 datasets are obtained, and the remaining 20 sets are used as test samples. The mean value of the training set is calculated, then this value is subtracted from the training set, and the training index set is used to build the LDA model. Since the label contains only two categories, the eight features will be reduced to one feature after dimensionality reduction, and this gives the comprehensive index sought in this article. The mapping vector obtained from this experimental data training process is:

(16) $$\omega ={\left(5013,3098.4,24.1599,22.173,-1555.6,243.2471,2.7641,5.3636\right)}^{T}$$

**Figure 18:**
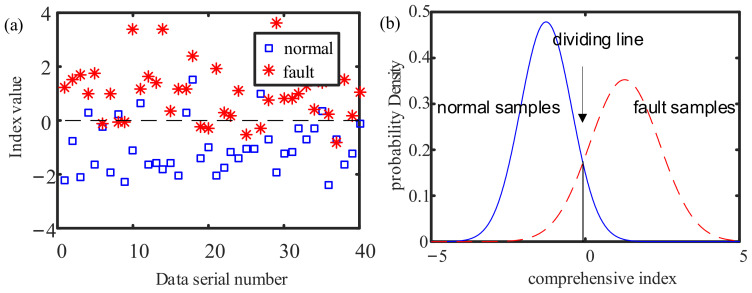
The result of dimensionality reduction for the training set: (**a**) comprehensive index; (**b**) probability density of comprehensive index.

The elements in the mapping vector represent the weights of the eight features in the comprehensive index. The result of dimensionality reduction for the training set is shown in Figure 18a, and the density function of the comprehensive index of the training set is shown in Figure 18b.

The comprehensive index shown in Figure 18a takes 0 as the dividing line; a value less than 0 is normal, and a value greater than 0 represents failure. It can be seen from the density function in Figure 18b that the average comprehensive index of the normal samples is −0.97, and the average comprehensive index of the fault samples is 1.02. The intersection area of the probability density curves for the normal samples and the fault samples is small. The normal and faulty samples can be more effectively distinguished by the dividing line taking 0 as the comprehensive index.

**Figure 19:**
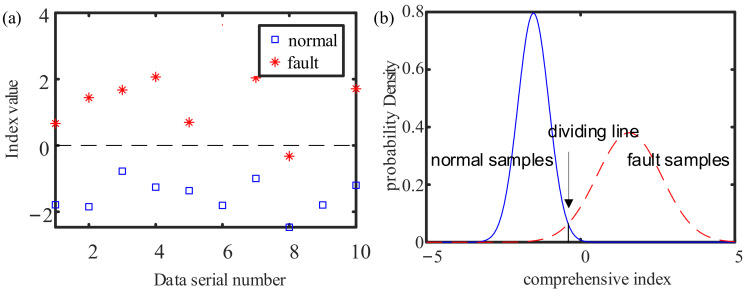
The result of dimensionality reduction for the test set: (**a**) comprehensive index; (**b**) probability density of comprehensive index.

According to the above model, a comprehensive index calculation is performed on the remaining test samples, and the result obtained is shown in Figure 19.

In Figure 19a, the rest of the test set is classified as normal, except for one fault datum, which is misjudged as normal. That is to say, the classification accuracy rate of the comprehensive index is 95%, which thus enables better operation status evaluation. The distribution range of the fault samples in the test set is relatively wide, and that of the normal samples is relatively concentrated, as shown in Figure 19b. Compared with the training set, the distribution function of the comprehensive index of the samples of the test set has a smaller intersectional area, which grounds better classification. These results confirm the effectiveness of the method proposed in this paper.

## 4. Conclusions

In this article, a three-phase current tacholess envelope order analysis method is proposed to solve the problem of feature extraction for a planetary gearbox under variable speed conditions. Firstly, the tacholess instantaneous rotation speed is estimated using three-phase current signals. Secondly, the order of the demodulated current signal is analyzed. Then, feature extraction is carried out for the envelope order signal. Finally, multiple features are fused into a comprehensive index using linear discriminant analysis to complete state feature extraction for the variable-speed planetary gearbox. The simulation analysis and experimental verification results show that the tacholess rotation speed estimation method proposed in this manuscript is effective in extracting instantaneous speed and performing fault diagnosis. Compared with the traditional time–frequency ridge extraction method, the tacholess speed estimation method proposed in this manuscript can improve the instantaneous speed estimation accuracy. The comprehensive index of envelope order proposed in this manuscript permits planetary gearbox state identification, and a 95% classification accuracy rate is achieved.

## Figures and Tables

**Table 1:** Order characteristic frequency of the planetary gearbox.

Characteristic Frequency	Meshing Frequency	Sun Gear	Planetary Gear	Ring Gear
Distributed Fault	24	0.67	1.34	0.34
Centralized Fault		2.04	2.68	1.02

## Data Availability

Data sharing is not applicable to this article.

## References

[1] Barszcz T., Randall R.B. (2009). Application of spectral kurtosis for detection of a tooth crack in the planetary gear of a wind turbine. Mech. Syst. Signal Process..

[2] Lewicki D.G., LaBerge K.E., Ehinger R.T., Fetty J. Planetary gearbox fault detection using vibration separation techniques. Proceedings of the 67th Annual Forum and Technology Display.

[3] Lei Y., Han D., Lin J., He Z. (2013). Planetary gearbox fault diagnosis using an adaptive stochastic resonance method. Mech. Syst. Signal Process..

[4] Feng Z., Qin S. (2016). Planetary gearbox fault diagnosis based on Hilbert vibration decomposition and higher order differential energy operator. J. Vib. Shock.

[5] Hong L., Dhupia J.S. (2014). A time domain approach to diagnose gearbox fault based on measured vibration signals. J. Sound Vib..

[6] Liu Q., Yang J. Fault diagnosis of wind turbine gearbox based on dual-tree complex wavelet and information entropy. Proceedings of the 2018 Prognostics and System Health Management Conference (PHM-Chongqing).

[7] Jiang P., Cong H., Wu C., Feng F. (2019). Research on feature enhancement method of planetary gearbox fault signal based on DWT-LPP. J. Acad. Armored Force Eng..

[8] Li H., Zhao J., Liu J., Ni X. (2016). Application of empirical mode decomposition and Euclidean distance technique for feature selection and fault diagnosis of planetary gearbox. J. Vibroeng..

[9] Si J., Cao Y., Shi X. Fault diagnosis of wind turbine planetary gear box based on EMD and resonance remodulation. Proceedings of the 2017 12th International Conference on Computer Science and Education (ICCSE).

[10] Shi X., Li H., Zhu X., Cao Y. Vibration fault diagnosis method for planetary gearbox of wind generating set based on EEMD. Proceedings of the 2019 14th International Conference on Computer Science and Education (ICCSE).

[11] Lei Y., Lin J., Zuo M.J., He Z. (2014). Condition monitoring and fault diagnosis of planetary gearboxes: A review. Measurement.

[12] Vicuña C.M. (2014). Effects of operating conditions on the acoustic emissions (AE) from planetary gearboxes. Appl. Acoust..

[13] Cardoso A.M., Saraiva E.S. (1993). Computer-aided detection of airgap eccentricity in operating three-phase induction motors by Park’s vector approach. IEEE Trans. Ind. Appl..

[14] Benbouzid M.E.H. (2000). A review of induction motors signature analysis as a medium for faults detection. IEEE Trans. Ind. Electron..

[15] Henao H., Kia S.H., Capolino G.-A. (2011). Torsional-vibration assessment and gear-fault diagnosis in railway traction system. IEEE Trans. Ind. Electron..

[16] Zhang J., Dhupia J.S., Gajanayake C.J. Model based current analysis of electrical machines to detect faults in planetary gearboxes. Proceedings of the 2014 IEEE/ASME International Conference on Advanced Intelligent Mechatronics.

[17] Zhang J., Dhupia J.S., Gajanayake C.J. (2015). Stator current analysis from electrical machines using resonance residual technique to detect faults in planetary gearboxes. IEEE Trans. Ind. Electron..

[18] Shi X., Li K., Du H., Si J. Research on fault diagnosis method for planetary gear of wind turbine generator based on MCSA and EMD. Proceedings of the 2017 IEEE 7th Annual International Conference on CYBER Technology in Automation, Control, and Intelligent Systems (CYBER).

[19] Cruz S.M.A., Cardoso A.J.M. (2000). Rotor cage fault diagnosis in three-phase induction motors by extended Park’s vector approach. Electr. Mach. Power Syst..

[20] Pires V.F., Martins J., Pires A., Rodrigues L. Induction motor broken bar fault detection based on MCSA, MSCSA and PCA: A comparative study. Proceedings of the 2016 10th International Conference on Compatibility, Power Electronics and Power Engineering (CPE-POWERENG).

[21] Sharma V., Parey A. (2020). Extraction of weak fault transients using variational mode decomposition for fault diagnosis of gearbox under varying speed. Eng. Fail. Anal..

[22] He G., Ding K., Li Y. (2016). Order tracking analysis for wind turbine planetary gearbox vibration based on meshing frequency and spectrum correction. J. Vib. Eng..

[23] Combet F., Gelman L. (2007). An automated methodology for performing time synchronous averaging of a gearbox signal without speed sensor. Mech. Syst. Signal Process..

[24] Wang Y., Xu G., Luo A., Liang L., Jiang K. (2016). An online tacholess order tracking technique based on generalized demodulation for rolling bearing fault detection. J. Sound Vib..

[25] Etien E., Allouche A., Rambault L., Doget T., Cauet S., Sakout A. (2021). A tacholess order analysis method for PMSG mechanical fault detection with varying speeds. Electronics.

[26] Lu S., Yan R., Liu Y., Wang Q. (2019). Tacholess speed estimation in order tracking: A review with application to rotating machine fault diagnosis. IEEE Trans. Instrum. Meas..

[27] Khazaee M., Ahmadi H., Omid M., Banakar A., Moosavian A. (2013). Feature-level fusion based on wavelet transform and artificial neural network for fault diagnosis of planetary gearbox using acoustic and vibration signals. Insight-Non-Destr. Test. Cond. Monit..

[28] Strączkiewicz M., Barszcz T. (2016). Application of artificial neural network for damage detection in planetary gearbox of wind turbine. Shock Vib..

[29] Yu J., Xu Y., Liu K. (2019). Planetary gear fault diagnosis using stacked denoising autoencoder and gated recurrent unit neural network under noisy environment and time-varying rotational speed conditions. Meas. Sci. Technol..

[30] Kang H., Luan J., Tian Y., Zheng H., Cao J. (2006). Application of the order tracking analysis in gear wearing. J. Vib. Shock.

[31] Wang Y., Xu G., Zhang Q., Liu D., Jiang K. (2015). Rotating speed isolation and its application to rolling element bearing fault diagnosis under large speed variation conditions. J. Sound Vib..

[32] Feng Z., Zhao L., Chu F. (2013). Vibration spectral characteristics of localized gear fault of planetary gearboxes. Proc. CSEE.

[33] Suo L., Liu F., Xu G., Wang Z., Yan W., Luo A. Improved park’s vector method and its application in planetary gearbox fault diagnosis. Proceedings of the 2018 IEEE International Conference on Prognostics and Health Management (ICPHM).

